# Involvement of the *Bacillus* SecYEG Pathway in Biosurfactant Production and Biofilm Formation

**DOI:** 10.1155/2024/6627190

**Published:** 2024-05-02

**Authors:** Frédéric Yannick Okouakoua, Christian Aimé Kayath, Saturnin Nicaise Mokemiabeka, David Charles Roland Moukala, Moïse Doria Kaya-Ongoto, Etienne Nguimbi

**Affiliations:** ^1^Laboratoire de Biologie Cellulaire et Moléculaire (BCM), Faculté des Sciences et Techniques, Université Marien N'GOUABI, BP. 69, Brazzaville, Congo; ^2^Institut National de Recherche en Sciences Exactes et Naturelles (IRSEN), Avenue de l'Auberge Gascogne, B.P 2400, Brazzaville, Congo

## Abstract

With *Bacillus* species, about 30% of extracellular proteins are translocated through the cytoplasmic membrane, coordinated by the Sec translocase. This system mainly consists of the cytoplasmic ATPase SecA and the membrane-embedded SecYEG channel. The purpose of this work was to investigate the effects of the SecYEG export system on the production of industrial biomolecules, such as biosurfactants, proteases, amylases, and cellulases. Fifty-two isolates of *Bacillus* species were obtained from traditional fermented foods and then characterized using molecular microbiology methods. The isolates secreted exoenzymes that included cellulases, amylases, and proteases. We present evidence that a biosurfactant-like molecule requires the SecA ATPase and the SecYEG membrane channel for its secretion. In addition, we showed that biomolecules involved in biofilm formation required the SecYEG pathway. This work presents a novel seven-target fragment multiplex PCR assay capable of identification at the species level of *Bacillus* through a unique SecDF chromosomal gene. The bacterial membrane protein SecDF allowed the discrimination of *Bacillus subtilis*, *B. licheniformis*, *B. amyloliquefaciens,* and *B. sonorensis*. SecA was able to interact with AprE, AmyE, and TasA. The Rose Bengal inhibitor of SecA crucially affected the interaction of AprE, AmyE, TapA, and TasA with recombinant Gst-SecA. The Rose Bengal prevented *Bacillus* species from secreting and producing proteases, cellulases, amylases, and biosurfactant-like molecules. It also inhibited the formation of biofilm cell communities. The data support, for the first time, that the SecYEG translocon mediates the secretion of a biosurfactant-like molecule.

## 1. Introduction

Protein export from the cytoplasm to the extracellular environment occurs in all living cells. SecYEG is an essential, ubiquitous, and universal export machinery for most proteins that integrate or translocate through the plasma membrane [1]. The SecYEG complex constitutes a central channel through which the unfolded substrate polypeptide chains are translocated under the control of the chaperone SecB [2]. In bacteria, SecYEG is composed of membrane proteins SecY, SecE, and SecG. More than one-third of newly synthesized ribosome proteins are translocated through this protein-conducting channel [3, 4]. The Sec exportome coordinates the secretion of polypeptides as preproteins that have fused cleavable signal peptides to the exported mature domains [1]. The integral membrane protein complex SecDF enhances protein translocation across the membrane driven by the complex of SecA ATPase and SecYEG [5, 6]. The products of the *secD* and *secF* genes form another heterotrimeric membrane complex, SecDF-yajC, which interacts with the SecYEG complex [7–10]. Bacterial secretory proteins are known to perform several essential “remote control” functions, such as food supply, cell-to-cell communication, and biofilm formation [3, 11–13]. It is therefore important to note that with the evolution and selection of bacterial species, many features remain unknown in terms of the SecYEG export component. The export process could target other molecules. To address this issue, we asked whether the SecYEG pathway can export a biosurfactant-like molecule.

Although mutants have been described in the literature regarding SecYEG components [10, 14–16], we use a different approach based on protein-protein interactions for the SecA proteins of the SecYEG system, TasA and TapA [12]. They are involved in the formation of biofilms and secretion of AmyE and AprE, which represent, respectively, amylase [17] and proteases [18–22]. For this work, recombinant proteins were fused with targeted proteins. The GST-pull-down assay then allowed for a systematic study of the interactome of interest.

In the present work, we show evidence that *Bacillus* spp. secrete biosurfactant-like molecules and form biofilms. We used SecDF, a proton-driven bacterial protein translocation factor, to discriminate isolates, and Rose Bengal was experimented to block the SecYEG pathway. We then tested the ability of SecA-SecYEG to export biosurfactant-like molecules and contribute to the formation of a biofilm. The motor protein SecA provides the driving force for translocation utilizing multiple ATP hydrolysis cycles. Rose Bengal is the first reported submicromolar inhibitor of this ATPase activity and protein translocation [6, 23–25]. No direct link between extracellular biosurfactants and SecYEG pathways has previously been clearly demonstrated. It is important to note that few studies have shown an involvement of the SecYEG pathway in the formation of biofilms. The present study is the first to demonstrate this involvement.

## 2. Materials and Methods

### 2.1. Sampling

Representative foods and beverages used in this study include Ntoba Mbodi (traditional fermented cassava leaves) and ginger wine (traditional fermented ginger tubers). 60 samples were collected from five markets located in different districts (Bacongo and Makélékélé) in Brazzaville (Republic of Congo).

### 2.2. Isolation and Characterization of Strains

10 g of each sample collected from fermented foods and beverages was aseptically sampled in a sterile tube. Using sterile physiological water, the sample was homogenized and distributed into ten sterile tubes. Dilutions were carried out, and bacterial suspensions were streaked on a Mossel agar medium (10.0 g peptone, 1.0 g of meat extract, 10.0 g of mannitol, 10% of egg yolk, 0.01 g of polymyxin B sulfate, 0.025 g of phenol red, 10.0 g of sodium chloride, 14.0 g of agar, and pH 7.2) for the growth of *Bacillus* species. The enumeration of colonies was performed in triplicate in each medium. The plates were incubated at 37°C for 24 h.

Most of the culture and biochemical tests were performed by using standard microbiological and biochemical methods. Each colony of Mossel agar having a distinct appearance was isolated separately. The purification of the isolates was performed rigorously by successive and alternating subcultures. The shape, size, and color of the bacterial colonies were recorded. The morphological characterization was performed using a light microscope (OPTIKA, Italy). Gram staining was performed using 3% potassium hydroxide (KOH) [26]. Speculation efficiency was assessed to determine the ability of isolates to form endospores [27, 28]. Briefly, bacterial cells cultured in TSB at 37°C for 24 were heat-inactivated. The number of spores was measured by measuring heat-resistant colonies (70°C for 30 min) in LB-agar plates, while viable cells were measured as total CFU in LB-agar plates. Spore % = (spores/mL)/(viable cells/mL) × 100%. The rapid test for detection of the cytochrome oxidase and catalase enzyme activity was performed for all bacterial strains according to the manufacturer guidelines (LIOFILCHEM, Italy). All purified isolated cultures were stored at −20°C in Lysogeny broth (LB) (10 g tryptone, 5 g yeast extract, and 10 or 5 or 0.5 g NaCl) containing 20% (v/v) glycerol.

### 2.3. Biomolecule Production

In order to investigate the viability activity of the SecYEG export systems in *Bacillus* strains, several tests were performed.

#### 2.3.1. Biosurfactant Production Assay

The emulsification activity was carried out using the method used by Uyar and Sağlam [29].

The emulsifying activity of a biosurfactant is its ability to retain an emulsion of hydrocarbons or oils in water. 5 mL of washed cells and 5 mL of acellular supernatant of each isolate were poured into a test tube containing 5 mL (v/v) of gasoline. The mixture was shaken vigorously for 3 min using a vortex mixer (VELP Scientifica, Italy). The tubes were then incubated at room temperature for 24 h. The height of the emulsion layer and the total height of the mixture were then measured. All the experiments were performed in triplicate, and the emulsification index (E24%) was calculated using the standard formula for E24% (**He**/**Ht**) × 100, with He being the emulsion height, Ht being the total height of mixture, and E24% being emulsification percentage after 24 h.

#### 2.3.2. Hydrolase Production Assay

*(1) Proteolytic Activity*. The ability of the isolates to produce proteases was based on their ability to degrade casein in skim milk. The methodology carried out was previously described [18].

Briefly, an overnight culture of *Bacillus* isolate was prepared in Luria broth (LB) at 37°C. The culture was then centrifuged at 12, 000 × *g* for 10 min, and 75 *µ*l of each culture supernatant was transferred to a well containing 1% agarose and skim milk (10%). The Petri dishes were incubated at 37°C for 24 h. The positive activity was revealed by the presence of a halo around the well; the diameters of the hydrolyzed halo were measured. The average of the three measurements was determined.

*(2) Amylolytic and Cellulolytic Activities*. Amylase and cellulase production was evaluated using cassava starch and cellulose as substrates. Briefly, 1 g of starch and 0.5 g of cellulose were added separately in 100 ml of LB agar, and the mixture was homogenized and sterilized in an autoclave at 121°C for 20 min. Then, the medium was poured into the Petri dishes and cooled to room temperature. A colony of each isolate was streaked on the media like a spot. Petri dishes were incubated at 37°C for 48 h, and a Lugol solution (Merck, Germany) was used to reveal positive activity. The average of the three measurements was taken [17]. The percentage of lysis for each isolated colony was evaluated using the formula %*L* = ((DT − DC)/DT), where %*L* is the percentage of lysis, DT is the total diameter (colony and halo), and DC is the colony diameter.

#### 2.3.3. Biofilm Formation Assay

The ability of the isolates to form biofilms was examined using the Congo Red (3,3′-([1,1′-biphenyl]-4,4′-diyl)bis(4-aminonaphthalene-1-sulfonic acid) agar and crystal violet (4-bis [4-(dimethylamino) phenyl] methylidene)-N, N-dimethylcyclohexa-2,5-dien-1-iminium chloride) assay. Briefly, 1.5 g of agar, 5 g of saccharose, Congo Red 0.008 g, and 2 g of LB were added to 100 mL of distilled water. The mixture was homogenized and sterilized in an autoclave at 121°C for 20 min. The medium was poured into the Petri dishes and cooled at room temperature. Each colony was streaked on the Congo Red agar. The Petri dishes were incubated at 37°C for 24 h. The positive activity was seen in colonies producing slime and turning black, as previously described [30]. The crystal violet assay was carried out as previously described by carrying out in 96-well MTP [31]. The average of the three measurements was taken.

#### 2.3.4. Effect of Rose Bengal (RB) on the Metabolism of *Bacillus* Strains

To confirm the link of the SecYEG export system to the production of biosurfactants, proteases, amylases, cellulases, and biofilm formation in *Bacillus* strains, Rose Bengal (GROSSERON, France) was used as an inhibitor of the SecA ATPase protein complex, as previously demonstrated [24]. In this study, Rose Bengal (4,5,6,7-tetrachloro-2′, 4′, 5′, 7′-tetraiodofluorescein) was used at concentrations ranging from 20 *µ*M to 100 *µ*M.

First, LB with or without Rose Bengal was prepared. Then, an overnight culture of each isolate was prepared in the two mediums at 37°C and the optical density at 600 nm was measured. The emulsification index, hydrolase production (cellulase, amylase, protease), and biofilm formation assays were performed using the Congo Red and crystal violet assay [31]. The average of the three measurements was taken.

### 2.4. Molecular Identification

#### 2.4.1. Genomic DNA Extraction

The primers for secDF amplification were designed using the NCBI portal (National Center for Biotechnology Information, https://www.ncbi.gov/Blast.cgi) using the genomic database of targeted strains such as *B. amyloliquefaciens*, *strains B. subtilis*, *B. licheniformis*, *B. altitudinis*, *B. mojavensis*, *B. safensis*, *B*. *sonorensis*, and *B. atrophaeus*. pDRAW32 software was used for bioinformatic analysis and check. The designed primers are shown in Table 1.

The extraction and purification of genomic DNA were performed according to the NucleoSpin Microbial DNA Kit (Macherey-NAGEL, Germany). Briefly, isolates were grown in 5 ml of LB for 24 h at 37°C with stirring. DNA purity was assessed by the ratio of UV absorbance (260/280 nm). 5 *μ*L of each amplification product was mixed with 2 *μ*L of loading buffer (BIOKÉ, the Netherlands). The mixes were subjected to electrophoresis in 1% agarose gel (w/v). The 10 kb 2-Log DNA sample (BIOKÉ, the Netherlands) was used as a molecular weight marker.

#### 2.4.2. SecDF Multiplex PCR Analysis

The identification of *Bacillus* species was carried out by the PCR multiplex using *secDF* genes from *Bacillus subtilis* subsp. subtilis str. 168 chromosome, complete genome (CP052842.1).

The amplification of *secDF* was used to identify *B. amyloliquefaciens*, *B. subtilis*, *B. licheniformis*, *B. altitudinis*, *B. mojavensis*, *B. safensis*, *B*. *sonorensis*, and *B. atrophaeus*. A forward primer (Bs-SecDF) was used (Bs-SecDF); all strains were discriminated with reverse primers (Bso-R, Bat-R, Bmo-R, Ba-R, Bs-R, BL-R, and Bae-R) (Table 1).

#### 2.4.3. Construction of Plasmids

Plasmid constructions and primers used in this work are listed in Table 2. The pGEX4T1 and pGEX6P1 plasmids (GE. Healthcare, France) were used to fuse in the frame glutathione S-transférase (GST) to *secA* or *tasA* or *tapB*. The pBAB-Myc-His plasmid (Thermo Fisher Scientific—Invitrogen, USA) was used to merge AmyE, aprE, tasA, and tapA in the frame (Table 2). PCR fragments were amplified from *B. subtilis* GL48, complete genome (CP052842.1) [32], by using OneTaq polymerase (BIOKÉ, the Netherlands).

### 2.5. Production of Recombinant Proteins and GST Pulldown Assay

*E. coli* strain BL21, harboring pGEX6P1 and pKIKO3 (pGEX6P-1-*secA*) and expressing glutathione S-transférase (GST) alone, and GST-SecA were cultured in LB with 100 *μ*g/mL ampicillin for 2 h at 37°C, and then, IPTG was added to a final concentration of 0.1 mM. After incubation for 3 h at 30°C, the bacteria were harvested and GST and GST-SecA were purified as described by the manufacturer of glutathione sepharose 4B (Amersham Pharmacia Biotech, UK). Briefly, GST-recombinant proteins were bound to glucose sepharose 4B (50 *μ*L). After fixing GST and GST-SecA on the resin, we mixed the fusion proteins with or without RB 50 *μ*M at room temperature for 1 h.

*E. coli* TOP10 strains, harboring pKIKO9 (pBAD-*aprE*-His), pKIKO10 (pBAD-*amyE*-His), pKIKO15 (pBAD-*tasA*-His), or pKIKO-tapA-His and expressing AprE-His, Amy-His and TasA-His, and TapA-His, were cultured in LB with 100 *μ*g/mL ampicillin for 2 h at 37°C, and then, arabinose was added to the cultures when they reached an OD_600_ of 0.6. After incubation for 3 h at 30°C, samples were mixed with cleared extract of *E. coli* TOP10 strains expressing AprE-His, AmyE-His, TasA-His, or TapA and incubated overnight at 4°C. The supernatants were removed after centrifugation, and the beads were washed five times with bead-binding buffer (1% Triton X-100 in 1X TBS solution), 1L (50 mM Tris-Cl, pH 7.4; 150 mM NaCl). After the final wash, an elution was performed with 40 *μ*L glutathione. The bound proteins were analyzed by electrophoresis in 12.5% polyacrylamide gels in the presence of SDS. Proteins were stained with Coomassie brilliant blue or transferred by electrophoresis to a polyvinylidene fluoride (PVDF) membrane for immunoblotting using anti-His (Sigma) or anti-GST antibodies (GE Healthcare, France).

pKIKO9, pKIKO10, pKIKO15, and pKIKO20 were introduced into *B. subtilis* by bacterial transformation. 0.001% arabinose was added to the cultures when they reached an OD_600_ of 0.6. Cultures were grown to an OD_600_ of 2, and culture supernatants were collected by centrifugation at 6, 000*g* for 15 min at 37°C. The supernatants were collected and precipitated with 4.5 g of ammonium sulfate ((NH4)_2_SO_4_) by overnight incubation at 4°C with shaking. The samples were collected and suspended in 200 *µ*L of 1X phosphate buffer saline (137 mM NaCl, 2.7 mM KCl, 10 mM Na_2_HPO_4_, and 1.76 mM KH_2_PO_4_) and then centrifuged at room temperature and analyzed by SDS-PAGE. Bound proteins were analyzed by immunoblotting using the anti-6-His antibody produced in rabbit (Sigma) and matrix-assisted laser desorption ionization-time of flight (MALDI-TOF VITEK, bioMérieux, Germany).

### 2.6. Determination of Enzymatic Secretion by Using Matrix-Assisted Laser Desorption Ionization-Time of Flight (MALDI-TOF)

Before detecting exogenous proteins, a 500-ml volume was centrifuged three times at 15, 000 × *g* for 15 min at 4°C, filtered through a 0.2-*µ*m pore size polytetrafluoroethylene (PTFE) membrane (Millipore), and then concentrated using a Millipore concentrator with a cutoff threshold of 5 kDa. After obtaining the concentrate sample, 100 *µ*L of culture supernatant of *Bacillus* species culture supernatant was spread in Petri dishes to control the presence of residual bacteria. Colorimetric protein determination with Coomassie blue (BioRad Bradford Assay) was used to quantify the amount of protein in the samples. The final volume (2.5 to 5 ml) was assessed for MALDI-TOF analysis. Sequences have been obtained and analyzed using the NCBI platform (https://www.ncbi.nlm.nih.gov/) and BLASTp (https://blast.ncbi.nlm.nih.gov/Blast.cgi?PAGE=Proteins).

### 2.7. Generating Polyclonal Antibodies and Bacillus Immunodetection

The GST-TasA and Gst-TapA fusion proteins constructed in pKIKO4 (pGEX4T1-TasA) and pKIKO5 (pGEX4T1-TapA) (Table 2) were obtained from the supernatant of *E. coli* BL21. Thrombin (EC 3.4.21.5) was used to digest the fusion protein. Pure TasA and TapA proteins were evaluated for antibody production. Polyclonal antibodies were generated using BALB/c mice without specific pathogens from a five-week-old female. 10 *μ*g of TasA and TapA was injected into nave BALB/c mice in the first week. An intraperitoneal injection was performed two weeks later to elicit antibody production. Serum was exhausted against the background of crude extracts and culture supernatants of *E. coli* BL21 that produce GST alone. Pure overnight cultures prepared in the LB medium of *B. subtilis* transformed with pKIKO9 (pBAD-aprE-His), pKIKO10 (pBAD-amyE-His), pKIKO15 (pBAD-tasA-His), and pKIKO-tapA-His were obtained. The supernatants were mixed with 80% NH_4_SO_4_ at +4°C. The cultures were centrifuged at 6000 rpm for 1 h, and the pellets were collected with 500 *μ*L of PBS. SDS-PAGE was stained with Coomassie blue, and immunodetection was performed using polyclonal antibodies anti-TasA and anti-TapA variants.

### 2.8. Statistical Analysis

GraphPad Prism version 5 software was used to determine significance thresholds. Principal component analysis (PCA) was used to investigate biofilm formation using Congo Red or crystal violet assays. Before ordination, the strain abundance data were transformed to better meet the assumption of normality using ln (*x* + 1). In addition, a PCA of biofilm formation was conducted to determine the capacity of strains to form a biofilm. All analyzes were performed using CANOCO (Canonical Community Ordination, version 4.5) [33].

## 3. Results

### 3.1. Isolation and Characterization of Isolates

Colony isolation in the Mossel medium was used to show the presence of bacteria of the genus *Bacillus*. Fifty-two (52) isolates were obtained, screened, and purified from the raw material, including 30 isolates from Ntoba Mbodi and 22 isolates from ginger wine. The purified isolates were characterized macroscopically and microscopically (data not shown). Among the 52 isolates, 57.6% (30/52) colonies were round, and 42.3% (22/52) were oval. All isolates were Gram-positive bacteria that were motile, catalase-positive, and able to form spores. 20% of the colonies had a dry texture, 60% were creamy, and 20% were viscous. According to Bergey's manual, the morphological and biochemical characterization of the isolates were presumptively identified as *Bacillus* species. The 52 isolates were used for subsequent tests and molecular identification.

### 3.2. The Production of Biosurfactant

**Figure 1:**
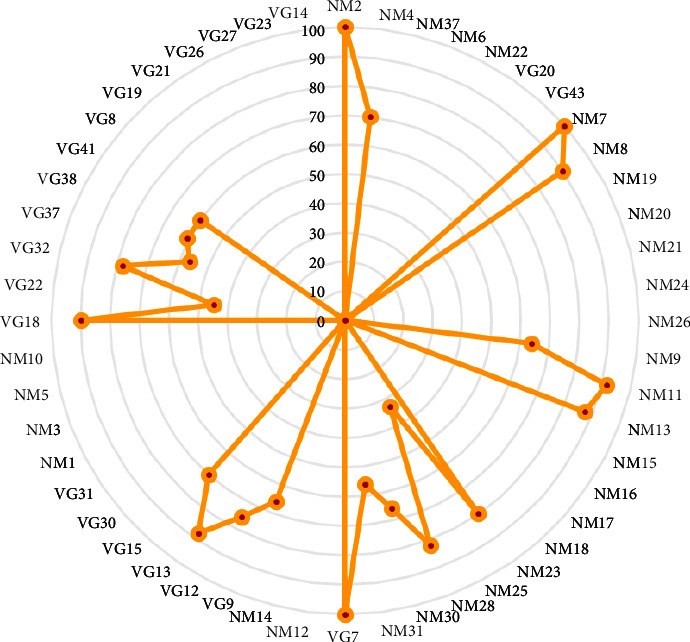
Fifty-two isolates were screened for their ability to produce biosurfactants. The emulsification index is shown for *Bacillus* isolates from VG: ginger wine and NM: Ntoba Mbodi.

To evaluate the production of biosurfactants, we determined the emulsification index (E24) of isolates using acellular supernatants. This study shows that 44.2% (23/52) of the isolates produced biosurfactant-like molecules (Figure 1) from supernatants having an emulsification index (E24) ranging from 33% to 100% (Figure 1). 15.3% (8/52) had the highest biosurfactant-like molecule production. This included NM2, NM7, NM8, NM11, NM13, VG7, VG13, and VG18. The emulsification index (E24) was ranging from 90% to 100% (Figure 1). 5.7% (3/52) of isolates (NM2, NM7, and VG7) had the highest percentage (100%) (Figure 1).

### 3.3. Hydrolase Production

**Figure 2:**
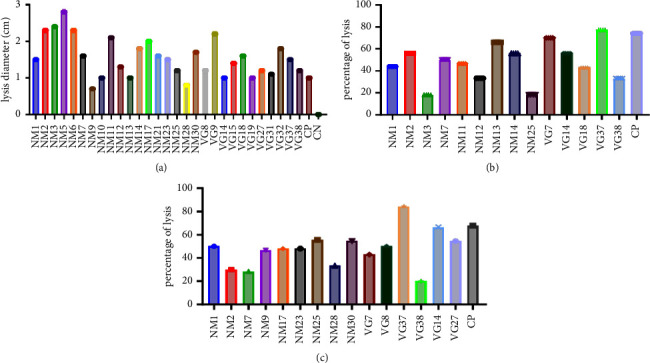
Production of hydrolases by *Bacillus* isolates. (a) Proteolytic activity (PA), (b) cellulolytic activity (CA), and (c) amylolytic activity (AA). CN: Negative control (*E. coli* BL21). CP: Positive control (*Bacillus subtilis* strain GL48 16S, GenBank: MK099888.1).

The ability of the isolates to produce hydrolases (proteases, cellulases, and amylase) is shown in Figures 2(a)–2(c), respectively. The study revealed that 55.8% (29/52) of the isolates were able to hydrolyze casein (Figure 2(a)), 26.9% (14/52) of the isolates hydrolyzed cellulose (Figure 2(b)), and 28.8% (15/52) hydrolyzed cassava starch (Figure 2(c)). 15.4% (8/52) of the isolates (NM1, NM2, NM7, NM25, VG7, VG14, VG37, and VG38) degraded all substrates (Figure 2). 11.5% (6/52) had the highest proteolytic activities (Figure 2(a)). This included NM2, NM3, NM5, NM6, NM9, and NM11. VG37 had the highest cellulolytic activity (Figure 2(b)). NM13, VG7, and VG37 had the highest amylolytic activity (Figure 2(c)). The diameter of the lysis ranged from 0.7 to 2.8 cm for the proteolytic activity; the percentage of hydrolysis ranged from 20 to 80% for the amylolytic and cellulolytic activity.

### 3.4. Biofilm Formation

**Figure 3:**
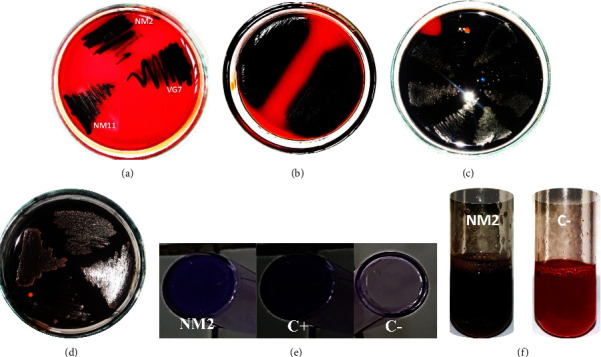
Biofilm formation by *Bacillus* isolates. (a) Black colony after 18 h of incubation, (b) diffusion on Congo Red agar after 24 h of incubation, (c) strong diffusion after 36 h of incubation, (d) complete diffusion after 48 h of incubation, (e) crystal violet assay. C+: *B. subtilis* strain GL48 used as a control in this study and C-: *E. coli* BL21 used as a negative control, (f) LB medium supplemented with Congo Red, NM2: isolates.

On the basis of proteolytic activity, the ability of 29 isolates to form biofilms was tested. We saw a variation in the phenotype of biofilm formation from one isolate to another. Concerning Congo Red, after incubation we observed a modification of the phenotype of the isolates that resulted in a diffusion of slime on the agar depending on the incubation period (Figures 3(a)–3(d)). The same phenotype was obtained when the formation of biofilms was evaluated on the LB medium supplemented with Congo Red (Figure 3). We also compared the capacity of *Bacillus* isolates to form biofilms using the Congo Red assay and crystal violet assay, which is the most sensitive method. As a result, 100% of the isolates were positive (Table 3), and 10.3% (3/29) were able to form a strong biofilm when Congo Red was used (NM23, NM2, and VG7). With crystal violet, 20.6% (6/29) strongly formed biofilms (NM23, NM2, VG7, NM37, VG37, and NM11). 13.8 (4/29) were moderate with Congo Red (NM11, NM37, VG37, and NM25). 0.3% (1/29) had a phenotype that did not change regardless of Congo Red or crystal violet (NM25). 20.6% (6/29) showed good results when tested with crystal violet. 34% (10/29) showed little biofilm formation, and 17.2% (5/29) were able to shift from low to moderate biofilm expression (NM4, NM7, NM26, VG8, and VG19) (Table 3).

### 3.5. Molecular Identification of Isolates Using PCR Multiplex Assay

The purified isolates were subjected to genomic DNA extraction. Seven primer-targeting genes encoding the SecDF protein complex were amplified and used to discriminate the *Bacillus* species. PCRs were performed to identify *Bacillus* species. The isolates VG38, VG7, and NM25 were identified as *B. subtilis*; VG37 and NM2 as *B.s licheniformis*; VG18 as *B. amyloliquefaciens*; and NM9 as *B. sonorensis*. The strains of *B. subtilis*, *B. licheniformis*, *B. amyloliquefaciens*, and *B. sonorensis* were used for the remainder of the work (Table 4).

### 3.6. MALDI-TOF Analysis

The *Bacillus* SecYEG pathway is capable of translocating certain exogenous proteins. To confirm the protein export through the SecYEG pathway, proteomic profiling of *B. subtilis*, *B. licheniformis*, *B. amyloliquefaciens*, and *B. sonorensis* was performed. The short peptide sequences were obtained from MALDI-TOF. Sequences were analyzed using BLASTp from the NCBI search platform (data not shown). On the basis of the MALDI-TOF sequence analysis, peptides were associated with AprE, SubC, AmyE (alpha-amylase), BglC, cellulase, and TasA. *The Bacillus* species that are capable of harboring and secreting the protein into the extracellular environment corresponded to *B. subtilis*, *B. licheniformis*, *B. amyloliquefaciens*, and *B. sonorensis*. These results indicate that the Sec-type secretion pathway is functional.

### 3.7. Effect of Rose Bengal

**Figure 4:**
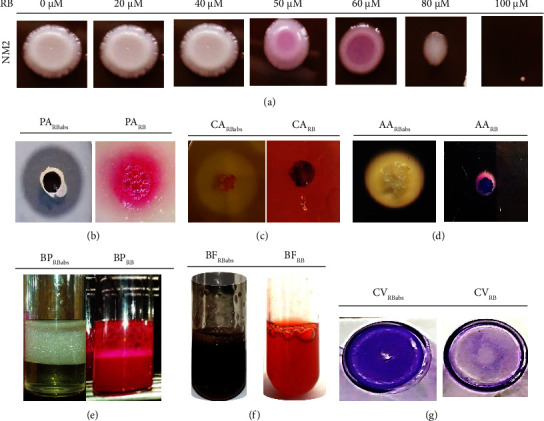
The effect of Rose Bengal on SecYEG-mediated secretion of biosurfactant and biofilm formation. (a) Determination of the concentration of Rose Bengal in the growth and morphology of isolates. NM2: *Bacillus* isolate; RB: Rose Bengal. (b) PA_RBabs_: Proteolytic activity in the absence of Rose Bengal and PA_RB_: proteolytic activity in the presence of Rose Bengal. (c) CA_RBabs_: Cellulolytic activity in the absence of Rose Bengal and CA_RB_: cellulolytic activity in the presence of Rose Bengal. (d) AA_RBabs_: Amylolytic activity in the absence of Rose Bengal and AA_RB_: amylolytic activity in the presence of Rose Bengal. (e) BP_RBabs_: Biosurfactant production in the absence of Rose Bengal and BP_RB_: biosurfactant production in the presence of Rose Bengal. (f) BP_RBabs_: LB biofilm production in the absence of Rose Bengal in LB and BP_RB_: LB biofilm production in the presence of rose Bengal in LB. (g) CV_RBabs_: Crystal violet assay of biofilm formation in the absence of Rose Bengal and CV_RB_: crystal violet assay of biofilm formation in the presence of Rose Bengal.

SecA is an ATPase that couples the hydrolysis of ATP to the release of bound precursor proteins enabling proton-motive-force-driven translocation across the cytoplasmic membrane. To illustrate the role and involvement of the SecYEG system in the biofilm formation mechanism and in the export and management of biomolecules produced by *Bacillus* species (*B*. *subtilis*, *B. licheniformis*, *B. amyloliquefaciens*, and *B. sonorensis*), Rose Bengal is used as an inhibitor of the SecA protein complex. The effective concentration of Rose Bengal was determined based on the bacterial morphology and metabolism at various concentrations (Figure 4(a)). The LB medium supplemented with Rose Bengal affected the morphology and metabolism of bacteria. Bacterial growth was normal at 20 to 50 *μ*M Rose Bengal with no significant difference (*P* > 005) compared to the absence of Rose Bengal. The half-maximum inhibitory concentration of Rose Bengal between 50 and 60 *μ*M was used to investigate all strains, including *B. subtilis*, *B. licheniformis*, *B. amyloliquefaciens*, and *B. sonorensis*. Beyond 50–60 *μ*M, the bacterial growth was altered.

Four *Bacillus* species were studied for their ability to form biofilms and secrete proteases, cellulases, amylases, and biosurfactants in the presence of Rose Bengal. At 50 *µ*M Rose Bengal, strains were unable to produce proteases (Figure 4(b)), cellulases (Figure 4(c)), and amylases (Figure 4(d)). An overnight culture of *B. subtilis*, *B. licheniformis*, *B. amyloliquefaciens*, and *B. sonorensis* was performed in the presence of Rose Bengal. All strains were unable to secrete biosurfactant-like molecules (Figure 4(e)). LB was supplemented with Congo Red and Rose Bengal (Figure 4(f)), and, interestingly, biofilm formation was inhibited characterized by the culture medium, which does not darken. The crystal violet assay was performed in the presence of Rose Bengal. This revealed the inhibition of biofilm formation in *B. subtilis*, *B. licheniformis*, *B. amyloliquefaciens,* and *B. sonorensis*. The dark blue appearance indicates the attachment of bacteria to the bottom surface of the microplates (Figure 4(g)).

### 3.8. Interaction between SecA, AprE, AmyE, TasA, and TapA in the Presence or Absence of Rose Bengal

**Figure 5:**
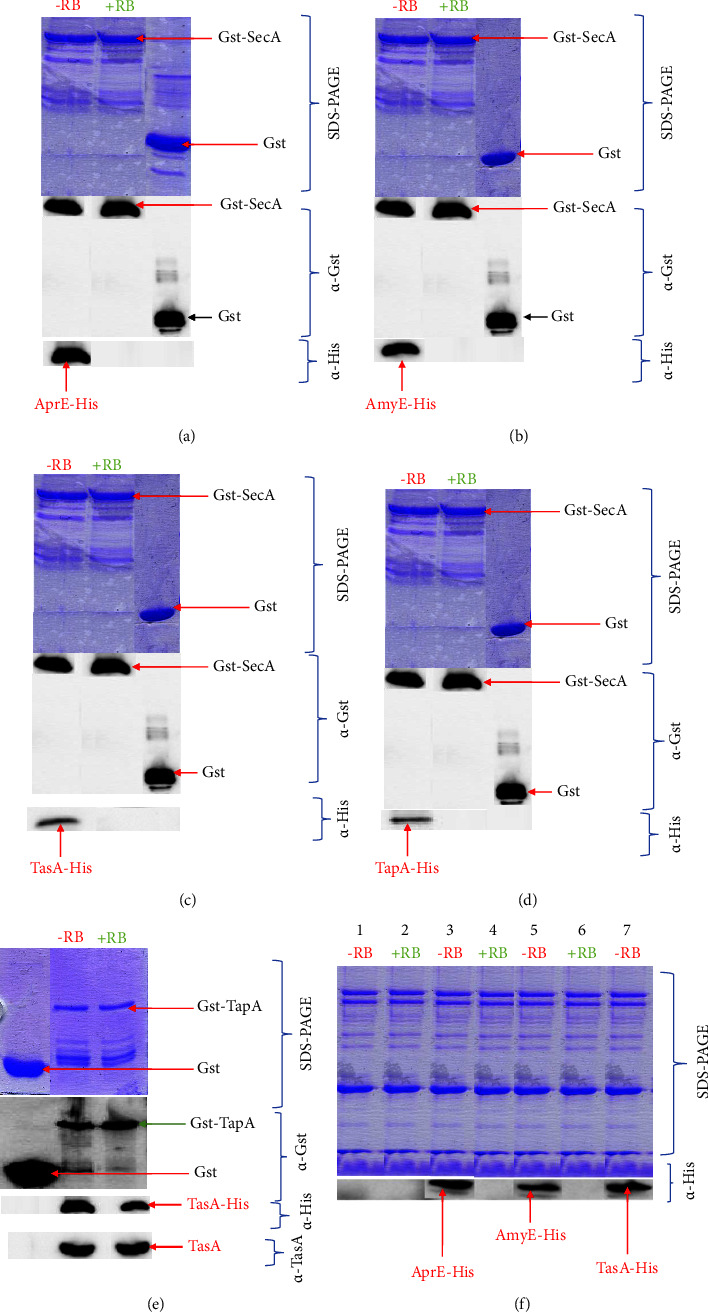
GST pulldown assay in the presence and absence of Rose Bengal. −RB: Absence of Rose Bengal; +RB: presence of Rose Bengal; GST: glutathione S-transferase. (a) Interaction between SecA and AprE, Gst-SecA: recombinant protein expressed in Gst fused in the frame with the SecA protein, and AprE-His: AprE encoding for subtilisin fused in the frame in the C-terminal region with the 6-His-tag. SDS-PAGE: sodium dodecyl sulfate–polyacrylamide gel electrophoresis, *α*-His: immunodetection using an anti-His, *α*-Gst: immunodetection using an anti-Gst. (b) Interaction between SecA and AmyE, AmyE-His: AmyE encoding amylase fused in the frame in the C-terminal region with the His-tag. (c) Interaction between SecA and TasA, TasA-His: TasA encoding for the biofilm component in the frame in the C-terminal region with the His-tag. (d) Interaction between Gst-SecA and TapA, TapA-His: TapA encoding for major biofilm matrix components in the frame in the C-terminal region with the His-tag. (e) Interaction between TapA and TasA in the presence or absence of Rose Bengal. Immunodetection using an anti-TasA, anti-His, and anti-Gst. (f) Extracellular protein from the culture supernatant. 1: *B. subtilis*: 2 and 3: *B. subtilis*/pKIKO9: strain expressing AprE-His in the presence or absence of Rose Bengal, 4 and 5: *B. subtilis*/pKIK10: strain that expresses AmyE-His in the absence or presence of Rose Bengal, and 6 and 7: *B. subtilis*/pKIKO15: strain that expresses Tas-His in the presence or absence of Rose Bengal.

We constructed plasmids pKIKO3, pKIKO9, pKIKO10, pKIKO15, and pKIKO20 that can, respectively, express the recombinant proteins GST-SecA, AprE-His, AmyE-His, TasA-His, and TapA to investigate the interaction between SecA and the excreted proteins AprE, AmyE, TasA, and TapA with and without Rose Bengal treatment (Figure 5). SecA interacted with AprE (Figure 5(a)) or AmyE (Figure 5(b)), TasA (Figure 5(c)), and TapA (Figure D) in the absence of Rose Bengal. The interaction was suppressed when Gst-SecA was first incubated with RB (Figures 5(a)–5(d)). Immunodetection using anti-GST and anti-His showed that antibodies recognized proteins qualitatively and quantitatively (Figures 5(a)–5(d)). Furthermore, we showed that TasA interacted with TapA (Figure 5(e)). The evidence was shown by using an anti-His and an anti-TasA (Figure 5(e)).

**Figure 6:**
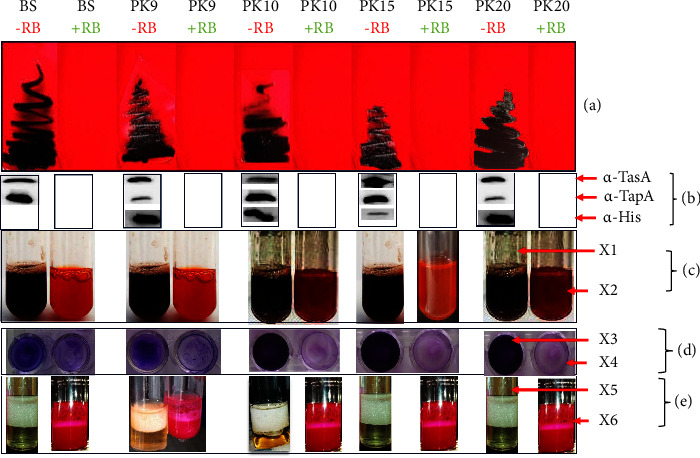
Effect of the inhibition of SecA by Rose Bengal (RB) on the ability to form biofilms and secrete biosurfactant-like molecules. −RB (red): Absence of Rose Bengal, +RB (green): presence of Rose Bengal. BS: *B. subtilis*, PK9: *B. subtilis* transformed with pKIKO9 expressing AprE-His, PK10: *B. subtilis* transformed with pKIKO10 expressing AmyE-His, PK15: *B. subtilis* transformed with pKIKO15 expressing TasA-His, PK20: *B. subtilis* transformed with pKIKO20 that expresses TapA-His. CR: Congo Red, CVA: crystal violet assay. LB: Lysogeny Broth. (a) LB agar supplemented with Congo Red in the presence or absence of RB. (b) Immunodetection using anti-TasA, anti-TapA, and anti-His antibodies. (c) LB supplemented with Congo Red in the presence or absence of Rose Bengal, *X*1: PK20 + LB + CR and *X*2 = PK20 + LB + CR + RB. (d) Crystal violet assay in the presence or absence of Rose Bengal, *X*3: PK20 in the absence of CVA and *X*4: PK20 in the presence of CVA. (e) Production of biosurfactant-like molecules in the presence or absence of Rose Bengal. *X*5: biosurfactant production in the absence of RB and *X*6: biosurfactant production in the presence of RB.

Plasmids pKIKO9, pKIKO10, pKIKO15, and pKIKO20 expressing, respectively, the recombinant proteins AprE-His, AmyE-His, TasA-His, and TapA-His were transformed into strains of *B. subtilis*, and we obtained, respectively, PK9, PK10, PK15, and PK20. We cultured them in the presence or in the absence of Rose Bengal. The constructions were made with the predicted signal peptides of the AprE-His, AmyE-His, and TasA-His proteins. *B. subtilis*, PK9, PK10, PK15, and PK20 were unable to form a biofilm on the LB agar supplemented with Congo Red in the presence of Rose Bengal (Figure 6(a)). We showed that proteins including AprE-His, AmyE-His, TasA-His, and TapA-His were indeed secreted into the extracellular environment, as shown by Western blotting using anti-His, anti-TasA, and anti-TapA (Figure 6(b)) in the absence of Rose Bengal. However, in the presence of Rose Bengal, *B. subtilis* and variants also lost their ability to secrete AprE-His, AmyE-His, TasA-His, TapA-His, TasA, and TapA (Figure 6(b)). Using MALDI-TOF technique, AprE, AmyE, TasA, and TapA proteins were identified, and short fragments were analysed (data not shown). In the presence of Rose Bengal, *B. subtilis* and variants were also phenotypically unable to form a biofilm by mixing strains with LB supplemented with Congo Red in the presence of Rose Bengal (Figure 6(c)). Furthermore, the biofilm formation capacity of *B. subtilis*, PK9, PK10, PK15, and PK20 strains was investigated by the crystal violet assay to evaluate the qualitative stages of the formation of bacterial biofilms. As a result, BS and variants were able to form a biofilm in the absence of Rose Bengal (Figure 6(d)). Finally, *B. subtilis*, PK9, PK10, PK15, and PK20 were unable to secrete biosurfactant-like molecules as explained in the methods (Figure 6(e)).

## 4. Discussion

The first findings described above indicate that *Bacillus* isolates secrete, in the extracellular environment, biosurfactant-like molecules and proteins of interest in biocatalysis such as proteases (AprE), amylases (AmyE), and cellulases. Proteases, amylases, and cellulases use the general secretion pathway because they have a signal sequence, in the N-terminal region, representing the postcard to their final destination. Enzymatic activities and Maldi-TOF shed light on these results. In prokaryotic and eukaryotic cells, it is important to remember that there are several known and unknown export systems. Many studies have been documented on this subject [3, 4]. Several secretion mechanisms have been clearly demonstrated. In the case of the SecYEG system in bacteria, we admit that this work provides added value in understanding the export of macromolecules. We postulate that this system would not be exclusively dedicated to proteins that have a clearly identified signal sequence. Moderation of this discussion offers a scientific and enlightened way to facilitate the understanding of this work.

The secretion of targeted macromolecules (cellulases, proteases, adenosine triphosphates, and biosurfactants) was demonstrated by using Rose Bengal. In addition to validating the secretion of extracellular molecules, our study used the Rose Bengal to block the SecA protein and therefore the SecYEG system. The role of the SecYEG translocon in protein secretion is well established, including descriptions of *B. subtilis*. The *Bacillus* genus is one of the more commercially interesting bacterial groups, generally recognized as safe, having the potential to produce cellulase [34], amylase [17], and proteases [18]. Studies of members of the *Bacillus* genus continue to surprise the scientific community. Our data suggesting that SecYEG mediates biosurfactant production are new although *Bacillus* species are known for their ability to produce biosurfactants [35]. For example, members of the genus produce various types of interesting biosurfactants, such as lipopeptides (surfactin and lichenysin) and glycolipids [36, 37]. It is conceivable that surfactin, the most studied biosurfactant, could use the SecYEG pathway. The rapid growth of bacteria of the *Bacillus* genus, quorum sensing, environmental adaptability, nutrient search, biofilm formation, and bacterial conjugation could be explanations that point in favor of the secretion of biosurfactants via the Sec translocase.

The new findings presented above show that SecDF could be used as a marker to discriminate *Bacillus* species. Our data support its use to discriminate *Bacillus* species such as *B. subtilis*, *B. licheniformis*, *B. amyloliquefaciens*, and *B. sonorensis*. DNA markers have been reported to discriminate *Bacillus* species in many studies [38–41].

Our finding shows that *Bacillus* isolates were able to produce biosurfactants. This is why Rose Bengal was used to drive this understanding and to demonstrate the link between SecYEG and biosurfactant secretion and biofilm formation. Rose Bengal and Eeyarestatin 24 (ES24) are known to inhibit SecYEG-mediated protein transport [24, 25, 42]. According to our understanding, Rose Bengal might interact with SecA or with SecDF [5, 10]. SecA is a key component of the Sec export system as it ensures the function of ATPase that produces the energy necessary to accomplish the export of extracellular protein from the cell. The ability of agents, such as Rose Bengal, to inhibit SecA ATPase has been reported [25]. The YerP protein has been implicated in the surfactin export [43]. *B. subtilis*, *B. licheniformis*, *B. amyloliquefaciens*, and *B. sonorensis* produce an important type of biosurfactant that includes lipoproteins and lipopeptides such as surfactin [44–47]. We also postulate that Rose Bengal treatment indirectly affects the surfactin export by affecting the function of YerP or related export proteins, such as YcxA and KrsE [43], which then prevent the YerP-surfactin interaction. Furthermore, the specificity of SecYEG could extend to interactions with surfactin, lichenysin, and glycolipids by using a scaffolding mechanism as well. *Bacillus* species are recognized as producers of lipopeptide or lipoprotein biosurfactants, such as surfactin. During the biosynthesis of extracellular biosurfactant, most of the peptides possess a predicted signal peptide for export [48]. Thus, additional work is needed to identify the proteins involved in the scaffolding mechanism.

In this work, we showed protein associations between SecA and proteins secreted in the extracellular environment (AmyE and AprE), on the one hand, and interactions with biofilm formation proteins (TapA and TasA), on the other hand. We also showed the interaction between TapA and TasA. These interactions clearly explain the functioning of the SecYEG system and also its role in the formation of biofilms. More studies are needed to detect other interactions. The SignalP6.0 version (https://services.healthtech.dtu.dk/services/SignalP-6.0/) shows that the prediction percentages of the signal peptide are 14.3%, 42.3%, 99%, and 99% for TapA, TasA, AprE, and AmyE, respectively. The low percentage of the TapA signal peptide could explain its interaction with TasA and therefore its export to the extracellular environment. On the string platform (https://string-db.org/), which is the predicted functional protein association network, YerP could interact with SecDF, YajC, YerQ, YerO, Yknx, YvrP, and YerP. The depletion of SecDF-YajC causes a decrease in the level of SecG, implying their functional interaction [7]. SecDF and YajC play an important role in the general SecYEG secretion pathway. This would bring them closer to the channel and allow the secretion of biosurfactant-like molecules into the extracellular area. We are not afraid to mention that our study has potential limitations. For example, we were unable to demonstrate direct interaction or non-interaction between surfactin and the components of SecYEG (SecA, SecB, SecDF, and YajC), on the one hand, and exogenous proteins that use the SecYEG pathway (AmyE, AprE, and Bgl), on the other hand. It is now important to investigate how SecDF and YajC could cooperate in the exportation of biosurfactant-like molecules.

Our latest findings show that the wild-type *B. subtilis* can easily express and secrete the AprE-His, AmyE-His, Tas-His, and TapA-His proteins into the extracellular environment using the SecYEG pathway. The presence of wild-type SecYEG confers the specificity for proper signal peptides, as reported here. The ability of the *Bacillus* genus to form biofilms is well described [30, 49]. A deficiency in surfactin production leads to a partial reduction in disease suppression by this biocontrol agent, which coincides with a defect in biofilm formation [50]. This suggests a close link between the components TasA and TapA, which are major components of the biofilm in *B. subtilis*. TapA and TasA were found as fibers in the biofilm matrix and are required for the structural integrity of the biofilm [11, 12].

## 5. Conclusions

Understanding the functioning of the Sec translocase is one of our major scientific challenges. This work adds a new scientific orientation in understanding the functioning of the general secretion pathway of macromolecules of both Gram-negative and Gram-positive bacteria. Coordination of protein transport is an essential keystone of the adaptation of bacteria to different environmental conditions. Bacteria will continue to surprise the scientific community, so it is unwise to assert that only proteins with a predicted secretion signal are expected to cross the barrier of the SecYEG or Sec61*αβγ* complex. The SecYEG or Sec61*αβγ* complex may prove to be necessary for the passage of a variety of macromolecules of interest, including activators, inhibitors, and/or quenchers. Its homology with Sec61*αβγ* opens perspectives in eukaryotic cells not only in cellular exchanges but also in cell-cell interactions. It should be noted that there are many human diseases affecting the gating of the Sec61*αβγ* complex. There are numerous cellular signal transductions that inevitably pass through the Sec61*αβγ* complex.

## Figures and Tables

**Table 1:** Sequences of *SecDF* primers used in this work.

Primers	Sequences (5′……3′)	Size (pb)	Target species
Bs-*Sec*DF-F	ATGAAAAAAGGACGCTTGATTGCGTTTTTC	1400	*B. subtilis*
Bs-R	CCCGGCAACCGTGACCGCACT		

Bs-*Sec*DF-F	ATGAAAAAAGGACGCTTGATTGCGTTTTTC	1140	*B*. *sonorensis*
Bso-R	TCTGACAGAGCTCGTACCAAAGATGAAC		

Bs-*Sec*DF-F	ATGAAAAAAGGACGCTTGATTGCGTTTTTC	589	*B. atrophaeus*
Bat-R	CATATTTAGGATTCTCTTTTTGCA		

Bs-SecDF-F	ATGAAAAAAGGACGCTTGATTGCGTTTTTC	900	*B. mojavensis,*
Bmo-R	GTCAAAAATCTGGAGTGTAATGTATACATAAACT		

Bs-SecDF-F	ATGAAAAAAGGACGCTTGATTGCGTTTTTC	1600	*B. amyloliquefaciens*
Ba-R	TTC ACG GAT CTT TTC TTT AGA TGG CTC TTCG		

Bs-SecDF-F	ATGAAAAAAGGACGCTTGATTGCGTTTTTC	840	*B. licheniformis*
BL-R	AGGCAAGCGATAGTAAAGCATAAATAA		

Bs-SecDF-F	ATGAAAAAAGGACGCTTGATTGCGTTTTTC	450	*B. aerophilus*
Bae-R	GCTTGATCGTCACGATCGGCTCATTTGTGT		

**Table 2:** Constructions, primers, and restriction enzymes used in this work.

Name of primers	Primer sequences (5′----3′)	Restriction enzymes	Size of PCR fragment (bp)	Characteristics	Given name	References
SecAs5	5′AGGATCCCTTGGAATTTTAAATAAAATGTTTGATCC-3′	BamHI	2523	pGEX6P-1-SecA	pKIKO3	This study
SecAAs5	5′TGTCGACCTATTCAGTACGGCCGCAGCAAT-3′	SalI				

TasASF TasAasF	5′TAGGATCCGGTATGAAAAAGAAATTGAG-3′ 5′TGTCGACTTAATTTTTATCCTCGCTATGCGCTTTTTC3′	BamHI–SalI	786	pGEX4T1-TasA	pKIKO4	This study

TapA-sF TapA-asF	5′TAGGATCCTTTCGATTGTTTCACAATCAGC-3′ 5′TGTCGACTTACTGATCAGCTTCATTGCTTTTTTC-3′	BamHI–SalI	762	pGEX4T1-TapA	pKIKO5	This study

AprE1 AprE2	5′TCTCGAGGTGAGAAGCAAAAAATTGTGGATCAG-3′ 5′AGGTACCTTGTGCAGCTGCTTGTACGTTGAT-3′	Xho1–KpnI	1146	pBAD-aprE-His	pKIKO9	This study

AmyE-s AmyE-As	5′TCTCGAGATGTTTGCAAAACGATTCAAAACCTC-3′ 5′AGGTACCATGGGGAAGAGAACCGCTTAAGCCCG-3′	Xho1–KpnI	659	pBAD-amyE-His	pKIK10	This study

TasA-TasA-As s	5′TCTCGAGATGGGTATGAAAAAGAAATTGAG-3′ 5′AGGTACCATTTTTATCCTCGCTATGCGCTTTTTC-3′	Xho1–KpnI	786	pBAD-TasA-His	pKIKO15	This study

TapA-S TapA-aS	5′TCTCGAGATGTTTCGATTGTTTCACAATCAGC-3′ 5′AGGTACCCTGATCAGCTTCATTGCTTTTTTC-3′	Xho1–KpnI	762	pBAD-TapA-His	pKIKO20	This study

**Table 3:** The ability of strains to form biofilm-like structures: comparison of Congo Red and crystal violet assays.

Isolate no.	Biofilm formation	
	Congo Red	Crystal violet assay
NM2	Biof (3+)	Biof (3+)
NM4	Biof (+1)	Biof (2+)
NM7	Biof (+1)	Biof (2+)
NM8	Biof (+1)	Biof (+1)
NM9	Biof (+1)	Biof (+1)
NM11	Biof (2+)	Biof (3+)
NM13	Biof (+1)	Biof (+1)
NM23	Biof (3+)	Biof (3+)
NM24	Biof (+1)	Biof (+1)
NM26	Biof (+1)	Biof (2+)
NM37	Biof (2+)	Biof (3+)
VG8	Biof (+1)	Biof (2+)
VG19	Biof (+1)	Biof (2+)
VG21	Biof (+1)	Biof (+1)
VG7	Biof (3+)	Biof (3+)
VG9	Biof (+1)	Biof (+1)
VG12	Biof (+1)	Biof (+1)
VG13	Biof (+1)	Biof (+1)
VG15	Biof (+1)	Biof (+1)
VG37	Biof (2+)	Biof (3+)
VG8	Biof (+1)	Biof (+1)
NM25	Biof (2+)	Biof (2+)

Biof (3+): strong biofilm formation. Biof (2+): moderate formation of Biofilm; Biof (+1): low biofilm formation.

**Table 4:** PCR multiplex identification of *Bacillus*.

Isolate no.	Species
NM9	*B*. *sonorensis*
Not identified among isolates	*B. atrophaeus*
Not identified among isolates	*B. mojavensis*
VG18	*B. amyloliquefaciens*
VG38, VG7, NM25	*B. subtilis*
VG37, NM2	*B. licheniformis*
Not identified among isolates	*B. aerophilus*

## Data Availability

The Excel sheets including the data used to support the findings of this study are available from the corresponding author upon request.

## References

[1] Oswald J., Njenga R., Natriashvili A., Sarmah P., Koch H. G. (2021). The dynamic SecYEG translocon. *Frontiers in Molecular Biosciences*.

[2] Diao L., Dong Q., Xu Z., Yang S., Zhou J., Freudl R. (2012). Functional implementation of the posttranslational SecB-SecA protein-targeting pathway in Bacillus subtilis. *Applied and Environmental Microbiology*.

[3] du Plessis D. J., Nouwen N., Driessen A. J. (2011). The Sec translocase. *Biochimica et Biophysica Acta, Biomembranes*.

[4] Cao T. B., Saier Jr M. H. (2003). The general protein secretory pathway: phylogenetic analyses leading to evolutionary conclusions. *Biochimica et Biophysica Acta, Biomembranes*.

[5] Vörös A., Simm R., Slamti L. (2014). SecDF as part of the Sec-translocase facilitates efficient secretion of Bacillus cereus toxins and cell wall-associated proteins. *PLoS One*.

[6] Hsieh Y. H., Huang Y. J., Zhang H. (2017). Dissecting structures and functions of SecA-only protein-conducting channels: ATPase, pore structure, ion channel activity, protein translocation, and interaction with SecYEG/SecDF•YajC. *PLoS One*.

[7] Kato Y., Nishiyama K., Tokuda H. (2003). Depletion of SecDF-YajC causes a decrease in the level of SecG: implication for their functional interaction. *FEBS Letters*.

[8] Nouwen N., Piwowarek M., Berrelkamp G., Driessen A. J. (2005). The large first periplasmic loop of SecD and SecF plays an important role in SecDF functioning. *Journal of Bacteriology*.

[9] Pogliano J. A., Beckwith J. (1994). SecD and SecF facilitate protein export in *Escherichia coli*. *The EMBO Journal*.

[10] Bolhuis A., Broekhuizen C. P., Sorokin A. (1998). SecDF of Bacillus subtilis, a molecular Siamese twin required for the efficient secretion of proteins. *Journal of Biological Chemistry*.

[11] Musik J. E., Zalucki Y. M., Day C. J., Jennings M. P. (2021). Expression of the Bacillus subtilis TasA signal peptide leads to cell death in *Escherichia coli* due to inefficient cleavage by LepB. *Biochimica et Biophysica Acta, Biomembranes*.

[12] Steinberg N., Keren-Paz A., Hou Q. (2020). The extracellular matrix protein TasA is a developmental cue that maintains a motile subpopulation within Bacillus subtilis biofilms. *Science Signaling*.

[13] Camara-Almiron J., Dominguez-Garcia L., El Mammeri N. (2023). Molecular characterization of the N-terminal half of TasA during amyloid-like assembly and its contribution to Bacillus subtilis biofilm formation. *NPJ Biofilms Microbiomes*.

[14] van der Wolk J., Klose M., Breukink E. (1993). Characterization of a Bacillus subtilis SecA mutant protein deficient in translocation ATPase and release from the membrane. *Molecular Microbiology*.

[15] Klein M., Hofmann B., Klose M., Freudl R. (1994). Isolation and characterization of a Bacillus subtilis secA mutant allele conferring resistance to sodium azide. *FEMS Microbiology Letters*.

[16] Takamatsu H., Nakane A., Oguro A., Sadaie Y., Nakamura K., Yamane K. (1994). A truncated Bacillus subtilis SecA protein consisting of the N-terminal 234 amino acid residues forms a complex with *Escherichia coli* SecA51(ts) protein and complements the protein translocation defect of the secA51 mutant. *Journal of Biochemistry*.

[17] Kayath C. A., Ibala Zamba A., Mokemiabeka S. N. (2020). Synergic involvements of microorganisms in the biomedical increase of polyphenols and flavonoids during the fermentation of ginger juice. *International Journal of Microbiology*.

[18] Faly S. M. A., Moyen R., Nguimbi E. (2017). Production, partial purification and based SDS-PAGE profiles of caseinolytic enzyme in two Bacillus strains isolated from fermented cassava leaves Ntoba mbodi in Congo Brazzaville. *Journal of Pure and Applied Microbiology*.

[19] Rajesh R., Gummadi S. N. (2022). Gummadi SN: alpha-Amylase and cellulase production by novel halotolerant Bacillus sp.PM06 isolated from sugarcane pressmud. *Biotechnology and Applied Biochemistry*.

[20] Dai J., Dong A., Xiong G. (2020). Production of highly active extracellular amylase and cellulase from Bacillus subtilis ZIM3 and a recombinant strain with a potential application in tobacco fermentation. *Frontiers in Microbiology*.

[21] Heng C., Chen Z., Du L., Lu F. (2005). Expression and secretion of an acid-stable *α*-amylase gene in Bacillus subtilis by SacB promoter and signal peptide. *Biotechnology Letters*.

[22] Shahhoseini M., Ziaee A. A., Ghaemi N. (2003). Expression and secretion of an alpha-amylase gene from a native strain of Bacillus licheniformis in *Escherichia coli* by T7 promoter and putative signal peptide of the gene. *Journal of Applied Microbiology*.

[23] Komarudin A. G., Driessen A. J. M. (2019). SecA-mediated protein translocation through the SecYEG channel. *Microbiology Spectrum*.

[24] Hsieh Y. H., Huang Y. J., Jin J. S. (2014). Tai PC: mechanisms of rose bengal inhibition on SecA ATPase and ion channel activities. *Biochemical and Biophysical Research Communications*.

[25] Cui J., Jin J., Hsieh Y. H. (2013). Design, synthesis and biological evaluation of rose bengal analogues as SecA inhibitors. *ChemMedChem*.

[26] Halebian S., Harris B., Finegold S. M., Rolfe R. D. (1981). Rapid method that aids in distinguishing Gram-positive from Gram-negative anaerobic bacteria. *Journal of Clinical Microbiology*.

[27] Lyu Y., Gu M., Chen M. (2019). Disruption of SpoIIID decreases sporulation, increases extracellular proteolytic activity and virulence in Bacillus anthracis. *Biochemical and Biophysical Research Communications*.

[28] Grossman A. D., Losick R. (1988). Extracellular control of spore formation in Bacillus subtilis. *Proceedings of the National Academy of Sciences of the United States of America*.

[29] Uyar E., Sağlam Ö (2021). Isolation, screening and molecular characterization of biosurfactant producing bacteria from soil samples of auto repair shops. *Archives of Microbiology*.

[30] Jebril N. M. T. (2020). Evaluation of two fixation techniques for direct observation of biofilm formation of Bacillus subtilis in situ, on Congo red agar, using scanning electron microscopy. *Veterinary World*.

[31] De Jesus R., Dedeles G. (2020). Data on quantitation of Bacillus cereus sensu lato biofilms by microtiter plate biofilm formation assay. *Data in Brief*.

[32] Kayath C. A., Ibala Zamba A., Goma-Tchimbakala J. (2019). Microbiota landscape of gut system of guppy fish (*Poecilia reticulata*) plays an outstanding role in adaptation mechanisms. *International Journal of Microbiology*.

[33] Ter Braak C. J. F., Šmilauer P. (2003). *Canoco 4*.

[34] Malik M. S., Rehman A., Khan I. U. (2023). Thermo-neutrophilic cellulases and chitinases characterized from a novel putative antifungal biocontrol agent: Bacillus subtilis TD11. *PLoS One*.

[35] Elenga-Wilson P. S., Kayath C. A., Mokemiabeka N. S., Nzaou S. A. E., Nguimbi E., Ahombo G. (2021). Profiling of indigenous biosurfactant-producing Bacillus isolates in the bioremediation of soil contaminated by petroleum products and olive oil. *International Journal of Microbiology*.

[36] Kaspar F., Neubauer P., Gimpel M. (2019). Bioactive secondary metabolites from Bacillus subtilis: a comprehensive review. *Journal of Natural Products*.

[37] Eras-Muñoz E., Farré A., Sánchez A., Font X., Gea T. (2022). Microbial biosurfactants: a review of recent environmental applications. *Bioengineered*.

[38] Felske A. D., Heyrman J., Balcaen A., de Vos P. (2003). Multiplex PCR screening of soil isolates for novel Bacillus-related lineages. *Journal of Microbiological Methods*.

[39] Ben-Dov E., Wang Q., Zaritsky A. (1999). Multiplex PCR screening to detect cry9 genes in Bacillus thuringiensis strains. *Applied and Environmental Microbiology*.

[40] Bourque S. N., Valero J. R., Mercier J., Lavoie M. C., Levesque R. C. (1993). Multiplex polymerase chain reaction for detection and differentiation of the microbial insecticide Bacillus thuringiensis. *Applied and Environmental Microbiology*.

[41] Kaya-Ongoto M. D., Kayath C. A., Nguimbi E. (2019). Genetic clearness novel strategy of group I Bacillus species isolated from fermented food and beverages by using fibrinolytic enzyme gene encoding a serine-like enzyme. *Journal of Nucleic Acids*.

[42] Schafer A. B., Steenhuis M., Jim K. K. (2023). Dual action of Eeyarestatin 24 on sec-dependent protein secretion and bacterial DNA. *ACS Infectious Diseases*.

[43] Tsuge K., Ohata Y., Shoda M. (2001). Gene yerP, involved in surfactin self-resistance in Bacillus subtilis. *Antimicrobial Agents and Chemotherapy*.

[44] Yao S., Gao X., Fuchsbauer N., Hillen W., Vater J., Wang J. (2003). Cloning, sequencing, and characterization of the genetic region relevant to biosynthesis of the lipopeptides iturin A and surfactin in Bacillus subtilis. *Current Microbiology*.

[45] Tsai W. C., Wong W. T., Hsu H. T. (2022). Surfactin containing Bacillus licheniformis-fermented products alleviate dextran sulfate sodium-induced colitis by inhibiting colonic inflammation and the NLRP3 inflammasome in mice. *Animals*.

[46] Feng R. Y., Chen Y. H., Lin C., Tsai C. H., Yang Y. L., Chen Y. L. (2022). Surfactin secreted by Bacillus amyloliquefaciens Ba01 is required to combat Streptomyces scabies causing potato common scab. *Frontiers in Plant Science*.

[47] Baindara P., Chowdhury T., Roy D., Mandal M., Mandal S. M. (2023). Surfactin-like lipopeptides from Bacillus clausii efficiently bind to spike glycoprotein of SARS-CoV-2. *Journal of Biomolecular Structure and Dynamics*.

[48] Hoffmann M., Mück D., Grossmann L. (2021). Surfactin from Bacillus subtilis displays promising characteristics as O/W-emulsifier for food formulations. *Colloids and Surfaces B: Biointerfaces*.

[49] Hussain M., Oh D. H. (2018). Impact of the isolation source on the biofilm formation characteristics of Bacillus cereus. *Journal of Microbiology and Biotechnology*.

[50] Zeriouh H., de Vicente A., Perez-Garcia A., Romero D. (2014). Surfactin triggers biofilm formation of *Bacillus subtilis* in melon phylloplane and contributes to the biocontrol activity. *Environmental Microbiology*.

